# HbA1c improvement in pregnant women with type 1 diabetes using the CamAPS FX automated insulin delivery (AID) system: Clinical and economic outcomes

**DOI:** 10.1111/dme.70394

**Published:** 2026-06-21

**Authors:** Maria‐Eleni Syleouni, Tara T. M. Lee, Federica Carrieri, Eleanor M. Scott, Aesha Khan Mirón, Helen R. Murphy

**Affiliations:** ^1^ Mylife Diabetes Care AG Burgdorf Switzerland; ^2^ Norwich Medical School University of East Anglia Norwich UK; ^3^ Department of Medicine and Aging Sciences G. d'Annunzio University of Chieti‐Pescara Chieti Italy; ^4^ Leeds Institute of Cardiovascular and Metabolic Medicine, School of Medicine University of Leeds Leeds UK; ^5^ Norfolk & Norwich University Hospital NHS Foundation Trust Norwich UK

**Keywords:** automated insulin delivery, CamAPS FX, clinical impact, economic impact, type 1 diabetes pregnancy, UK

## Abstract

**Aims:**

The Automated Insulin Delivery among Pregnant women with Type 1 diabetes (AiDAPT) trial demonstrated that using the CamAPS FX automated insulin delivery (AID) was associated with improved glycaemic outcomes. This study aimed to assess the clinical and economic impact of improving third‐trimester HbA1c.

**Methods:**

Using the AiDAPT data, a health economics model was built to estimate clinical outcomes and healthcare resource utilisation associated with third‐trimester HbA1c categories (<42 mmol/mol, 42–53 mmol/mol, ≥53 mmol/mol). AiDAPT HbA1c distribution and associated clinical outcomes (obstetric: pre‐eclampsia, delivery method; neonatal: length of stay in normal/critical/intensive care) were used to estimate perinatal complications. UK‐specific healthcare costs were extracted from the NHS National Schedule and other published sources. Total costs to the UK healthcare system were estimated using an incremental 3.3 mmol/mol (0.3%) HbA1c reduction observed with CamAPS FX AID over Standard Care in AIDAPT.

**Results:**

In the model, HbA1c improvement with CamAPS FX AID was associated with projected reductions in pre‐eclampsia risk (−13%), high dependency neonatal care days (−16%), neonatal intensive care unit days (−12%) and clinic visits. The model projected cost reduction for the NHS healthcare system of £6,696,636 under conservative modelling assumptions, being cost saving at 96.8% of the simulations. The projected incremental cost difference per woman treated with CamAPS FX AID was ‐£1016, mainly derived from a modelled shift in the distribution of neonatal care, with reduced length of stay in high dependency neonatal intensive care.

**Conclusion:**

CamAPS FX AID improves third‐trimester HbA1c with projected cost reductions for the UK healthcare system.

## Graphical abstract

Clinical and economic benefits of using CamAPS FX pregnancy‐specific AID system.

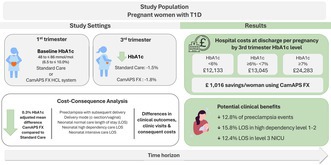

**What's new?**

- The CamAPS FX automated insulin delivery (AID) system was developed to address the gestational challenges of type 1 diabetes and support women to achieve optimal pregnancy outcomes. Yet evidence on its broader clinical and economic impact remains limited.
- This study demonstrates that the third‐trimester HbA1c improvement observed in AiDAPT with use of the CamAPS FX AID system was projected to reduce obstetric and neonatal complications, including maternal pre‐eclampsia and neonatal critical care needs.
- Using UK‐specific cost data, conservative analyses show projected cost savings of >£1000 per pregnancy, mainly driven by reduced neonatal intensive care.
- Our model‐based findings highlight that optimising maternal glucose with CamAPS FX AID could bring both clinical and economic value and inform NHS decision‐making on its widescale adoption across maternity clinics.

## 1 INTRODUCTION

Achieving and maintaining optimal maternal glucose levels during type 1 diabetes pregnancy is associated with reduced risk of pre‐eclampsia^1^ and neonatal complications, such as preterm births, large birthweight,^2^, ^3^ admission to neonatal intensive care unit (NICU) and perinatal death.^3^ UK population data indicate that entering the third trimester with HbA1c 48 mmol/mol (>6.5%) increases the risk of perinatal mortality (3‐fold increase).^2^ Thus, optimising second and third trimester glycaemic control is critical to reduce adverse maternal‐fetal outcomes and healthcare costs.

The CamAPS FX was the first commercially approved automated insulin delivery (AID) system for use during type 1 diabetes pregnancy.^4^ It was uniquely designed to address the dynamic physiological and clinical needs of pregnancy and is currently the only AID algorithm that incorporates pregnancy‐specific data during its development phase.^5^, ^6^ Moreover, the CamAPS FX AID system allows users to set ambitious personal glucose targets (4.5–5.0 mmol/L), as recommended during pregnancy.^7^

In the AiDAPT randomised controlled trial (RCT), a UK multicentre study, two methods of glucose optimisation, targeting pregnancy glucose targets, were compared in 124 participants: CamAPS FX AID versus Standard Care – (multiple daily injections [MDI] + continuous glucose monitoring [CGM] or continuous subcutaneous insulin infusion [CSII] + CGM). The results showed improved clinical efficacy for the primary outcome of time spent in pregnancy range (TIRp 3.5–7.8 mmol/L) and across key secondary outcomes, including mean glucose, time above pregnancy range (TARp >7.8 mmol/L), time above other hyperglycaemic thresholds (6.7 mmol/L, >10.0 mmol/L), overnight TIRp and HbA1c.^8^

The CRISTAL RCT, a multicentre study conducted in Belgium and the Netherlands involving 101 participants compared the Minimed 780G AID system versus Standard Care (CSII+CGM) during type 1 diabetes pregnancy, did not demonstrate significant between‐group differences in the primary outcome of percentage time in pregnancy range (TIRp 3.5–7.8 mmol/L) or in key glycaemic outcomes, including mean glucose, time above target range and Hba1c.^9^ There was a small difference (an extra 24 min) spent in the overnight TIRp 3.5–7.8 mmol/L between 24.00–06.00 h, which reached statistical significance but does not represent a minimally important clinical difference. Also, cohort studies reported no improvement in the glycaemic outcomes associated with using non‐pregnancy‐specific AID systems.^7^, ^10^ Suboptimal maternal and neonatal outcomes were observed,^7^, ^10^ including increased risk of delivering large‐for‐gestational‐age infants and higher rates of caesarean section.^10^

A recently published health economic evaluation conducted from the Spanish healthcare perspective reported the clinical and economic benefits of improved glycaemic control associated with the use of CamAPS FX AID during pregnancy.^11^ Another study from Belgium examined the cost‐effectiveness of using the Minimed 780G AID during T1D pregnancy.^12^ These authors used data from the CRISTAL trial, although no clinically relevant glycaemic improvements for key glycaemic outcomes were achieved,^9^, ^12^ potentially due to the limited pregnancy‐specific algorithm adaptation.

The primary objective of this study was to assess the modelled clinical benefits and potential cost savings associated with third‐trimester HbA1c improvement as observed with CamAPS FX AID use in the AiDAPT trial if applied across the UK population of pregnant women with type 1 diabetes.

Throughout this manuscript, although we use sexed language, referring to ‘pregnant women’ and ‘mothers’, study findings are inclusive of and relevant for all people who are pregnant or have given birth.^13^

## 2 METHODS

### 2.1 Study population

In this study, data from the AiDAPT randomised controlled trial on 124 pregnant women with type 1 diabetes from nine antenatal diabetes centres across England, Scotland and Northern Ireland were used.^8^ The AiDAPT trial participants were randomised to receive either Standard Care (CGM with MDI or insulin pump therapy) or the CamAPS FX AID system (ISRCTN56898625). Participants had a mean age of 31.1 years and were recruited at approximately 10–12 weeks' gestation. Early pregnancy HbA1c ranged from 43 to 129 mmol/mol (6.1%–14.0%), with a mean baseline HbA1c of 61 mmol/mol (7.7%). Participant characteristics such as age, baseline HbA1c levels, and duration of diabetes were similar to those of the UK National Pregnancy in Diabetes audit 2021–2023.^14^ For the current analysis, women who experienced pregnancy loss before 24 weeks were excluded, as the model evaluated obstetric and neonatal outcomes applicable to pregnancies of at least 24 weeks of gestation.

In AiDAPT, participants randomised to the CamAPS FX AID system demonstrated significantly greater improvements in HbA1c than those in Standard Care (mean adjusted difference: −0.31 percentage points; 95% Confidence Interval, −0.50 to −0.12) (Table 1). This was accompanied by favourable outcomes across most secondary glycaemic endpoints.^8^

**TABLE 1:** Population characteristics of AiDAPT participants (*N* = 124) and third‐trimester HbA1c level.

Characteristic	Value
Age (years), mean (SD)	31.1 (SD: 5.3)
Standard Care group (*N* = 63)	30.2 (SD: 5.5)
CamAPS FX group (*N* = 61)	32.0 (SD: 5.0)
White race‐ no (%) (SD)	
Standard Care group	57 (90%)
CamAPS FX group	58 (95%)
HbA1c %, mean at baseline (SD)	7.7% (SD: 1.2)
Standard Care group	7.9% (SD: 1.3)
CamAPS FX group	7.6% (SD: 1.1)
HbA1c %, mean at antenatal intervention phase (SD)	
Standard Care group	6.4% (SD: 0.5)
CamAPS FX group	6.0% (SD: 0.5)
HbA1c %, adjusted mean difference SC vs. CamAPS FX (CI)	−0.3% (−0.50 to −0.12)
Clinic visits	
Standard Care group	1.5
CamAPS FX group	1.1

Abbreviations: CI, 95% confidence interval; SD, standard deviation.

### 2.2 Health economics analysis

**FIGURE 1:**
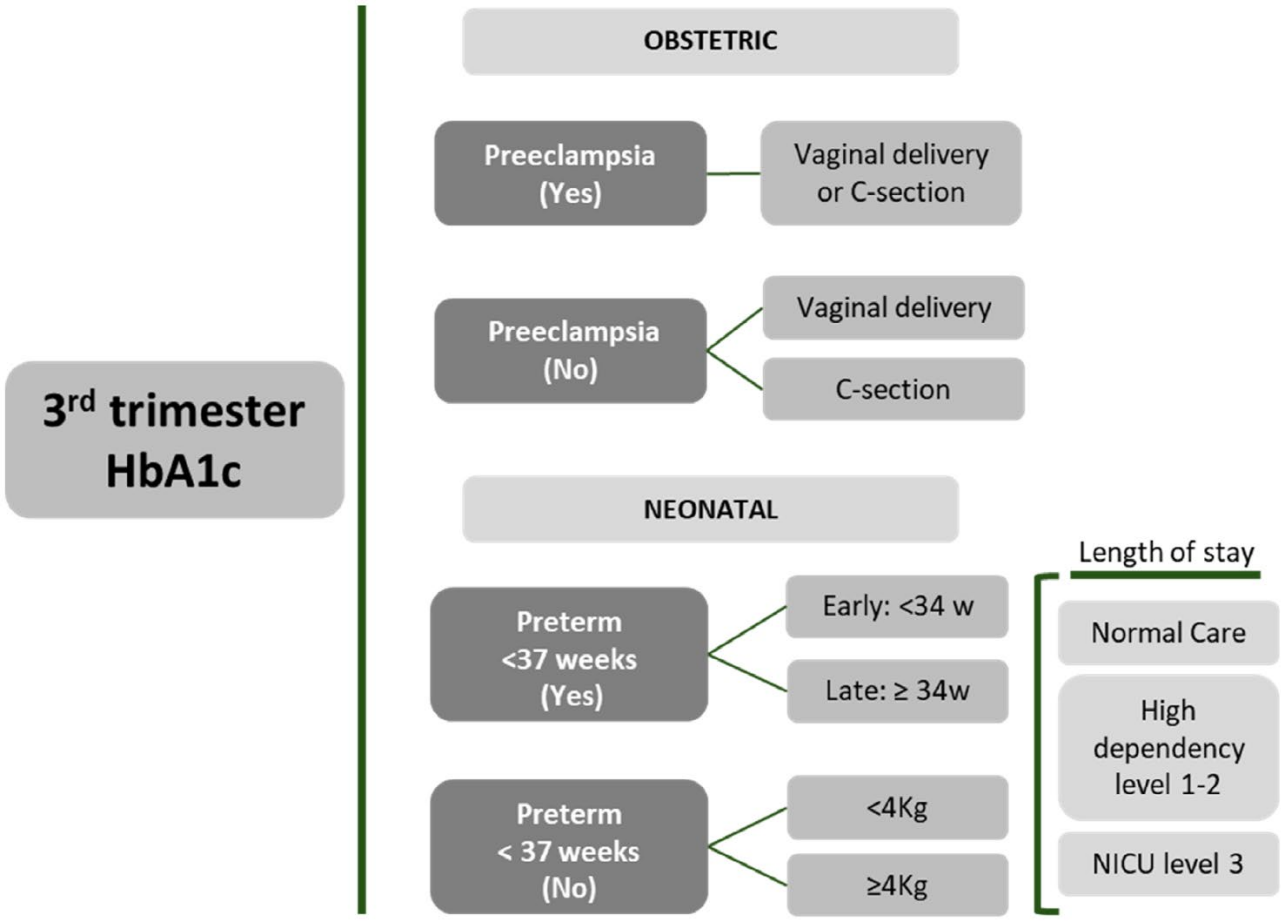
The decision‐analytic model structure linking third‐trimester HbA1c categories to maternal and neonatal outcomes. NICU: Neonatal intensive care unit.

This study assessed the modelled clinical and economic consequences of improved maternal glucose outcomes, reflected by HbA1c improvement (the primary modelling variable) between the first and third trimester. HbA1c was selected given its well‐established associations with serious adverse pregnancy outcomes,^2^, ^15^ its use in routine clinical practice and in clinical care pathways.^16^, ^17^ HbA1c was used as a summary measure to stratify pregnancy risk and is not intended to represent the full complexity of gestational glycaemic changes. The analysis employed a decision tree, with branches for both obstetric and neonatal outcomes, stratified by maternal HbA1c category (Figure 1). The following three HbA1c categories were observed in the data during the third trimester (≥28 weeks' gestation); HbA1c <42 mmol/mol (<6%), HbA1c 42–53 mmol/mol (6%–7%), HbA1c ≥53 mmol/mol (≥7%).

The observed HbA1c improvement was modelled to the first trimester (≤13 weeks and 6 days gestation) HbA1c distributions of all AiDAPT participants, shifting the proportion of women across HbA1c categories at the time of outcome ascertainment. Finally, all obstetric and neonatal outcome probabilities were derived from the resulting third‐trimester HbA1c distribution data of the AiDAPT cohort, reflecting the expected glycaemic profile under each treatment group.

### 2.3 Outcomes

This analysis considered obstetric outcomes: pre‐eclampsia with subsequent delivery, mode of delivery (vaginal delivery or caesarean section), and neonatal outcomes dependent on gestational age (preterm <37 weeks; term ≥37 weeks): mean length of stay in days in normal care, high dependency care (level 1–2), and neonatal intensive care (level 3).^18^ The neonatal care length of stay data, as described in AiDAPT, were assumed to capture the care associated with birth complications, including neonatal hypoglycaemia, premature birth, macrosomia and respiratory distress syndrome. Pre‐eclampsia was modelled as a maternal complication associated with subsequent delivery. For women with pre‐eclampsia, delivery was assumed as unassisted vaginal birth, assisted vaginal birth, elective and emergency caesarean section, based on data reported by Fox et al.^19^ These were used to estimate an average delivery cost for women with pre‐eclampsia, whereas among women without pre‐eclampsia, the mode of delivery was modelled explicitly as vaginal delivery or caesarean section with corresponding probabilities applied. Clinic visits were also included as a separate cost component and were not modelled as a function of third‐trimester HbA1c categories. Instead, these were estimated based on the follow‐up visits observed during AiDAPT, with unit costs derived from NHS reference costs. The following outcomes were excluded: neonatal death, miscarriage events, and readmission to the hospital after discharge.

While AiDAPT systematically collected all outcomes relevant to the present study under controlled settings, it was not statistically powered to show differences for all these outcomes. Finally, all clinical risk estimates as well as length of stay were treated as fixed parameters in the model. Therefore, parameter level uncertainty intervals were not estimated and uncertainty was instead explored through probabilistic sensitivity analysis.

### 2.4 Costs and time horizon

The economic impact analysis was performed from the UK healthcare system perspective, for pregnant women with type 1 diabetes using national data for the period 2021–2023. Over this period, 6590 pregnancies in women with type 1 diabetes were reported.^14^ The cost assessment focused on direct costs related to the immediate obstetric and neonatal outcomes at birth, comparing the Standard Care with CamAPS FX AID, as well as the additional clinic visits during the study period.

Costs related to diabetes management and any neonatal re‐admission were excluded from the analysis. Consequently, the estimates represent a conservative assessment of the direct costs associated with immediate obstetric and neonatal outcomes.

Most cost inputs used for this economic analysis were sourced from the NHS national schedule report.^20^ Costs related to vaginal delivery and caesarean section were estimated as weighted averages for the respective codes and distributions reported in the data. Cost for pre‐eclampsia was sourced from a study in Ireland^19^ and was assumed to be analogous to that of the United Kingdom. Finally, all cost parameters for this model were adjusted to 2024 GBP (£) to reflect current economic conditions and impact (Table 2), and were assumed to be fixed as the other model parameters. The article has been written following the ISPOR CHEERS checklist.

**TABLE 2:** Unit costs of obstetric and neonatal outcomes are used as model inputs. All cost values are inflated for 2024.

Outcomes	Costs (£)	References
Obstetric		
Pre‐eclampsia, including subsequent delivery*	£5972	Fox et al.^19^
C‐section	£7144	NHS England^14^
Vaginal delivery	£3242	NHS England^14^
Neonatal		
Normal care	£601	NHS England^14^
High dependency level 1–2	£1456	NHS England^14^
Neonatal intensive care unit	£2029	NHS England^14^
Clinic visit	£210	NHS England^14^

* Composite cost reflecting management of preeclampsia and subsequent delivery, based on Fox et al.^19^

### 2.5 Sensitivity analysis

To assess model uncertainty and the impact of participant allocation across HbA1c groups, a probabilistic sensitivity analysis was conducted using Monte Carlo simulations. Using 1000 iterations, a beta distribution was applied to vary the allocation of the participants to each third‐trimester HbA1c group under the CamAPS FX AID or the Standard Care groups. In each iteration, HbA1c‐specific clinical outcome probabilities and unit costs were held constant, such that the probabilistic sensitivity analysis reflects uncertainty in HbA1c category allocation rather than full parameter uncertainty across all model inputs. Uncertainty was summarised using the 2.5th and 97.5th percentiles of the simulated distribution and is reported as 95% uncertainty intervals, representing the central 95% of modelled outcomes. A deterministic one‐way sensitivity analysis with ±20% variation was performed individually for all cost inputs, while holding all other parameters at their base‐case values.

### 2.6 Ethics

In this study, all input parameters were sourced from the previously published AiDAPT study protocol, which included the health economic evaluation.^18^

## 3 RESULTS

### 3.1 Base case analysis

The estimated healthcare costs for the immediate obstetric and neonatal outcomes, as well as clinic visits for all T1D pregnant women with singleton births in the United Kingdom (2021–2023) under the Standard Care treatment were estimated at £94,526,886 (95% uncertainty interval: £90,418,213 to £98,438,642). When alternative treatment with CamAPS FX AID was considered, then the modelled obstetric and neonatal outcomes costs, as well as clinic visits, in relation to these outcomes were estimated to decrease to £87,830,250 (95% Uncertainty interval: £84,969,191 to £90,571,075). The modelled cost reductions for the UK healthcare system were estimated to be at least £6,696,636 (95% Uncertainty interval: −£8,335,085 to £732,921). CamAPS FX AID was projected to be cost saving at 96.8% of the simulations. The projected incremental cost difference per woman treated with CamAPS FX AID was −£1016 with 95% uncertainty Interval ranging from −£1265 to £111 (negative values indicate cost savings). This was predominantly attributed to the shift in the distribution of the neonatal care, namely, reduced length of stay in high dependency neonatal intensive care units.

**FIGURE 2:**
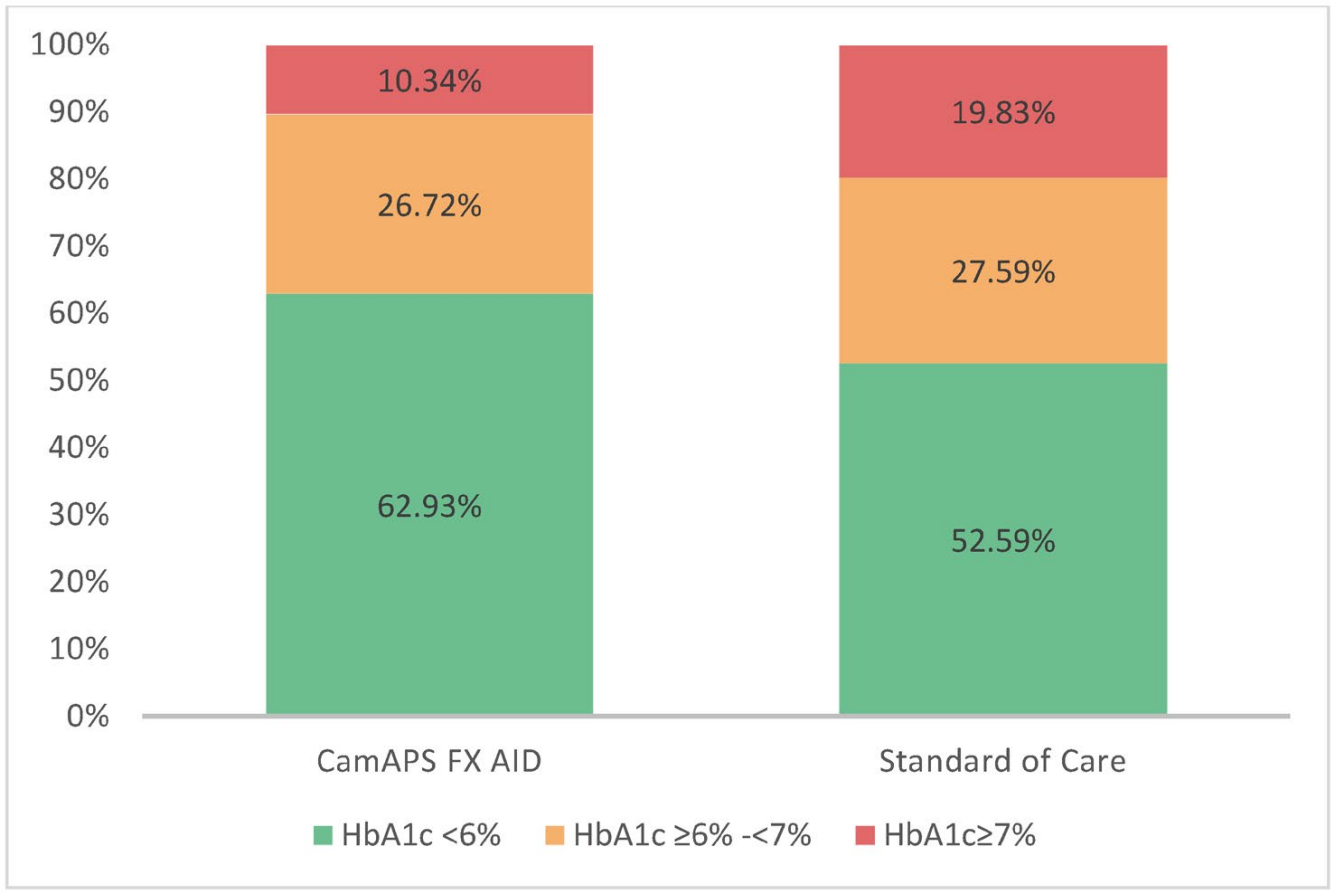
Third‐trimester HbA1c distributions by treatment group: CamAPS FX and Standard Care.

Overall, the total healthcare system costs were influenced by the third‐trimester HbA1c distribution across each treatment group (Figure 2). With CamAPS FX AID, 90% of women were projected to achieve an HbA1c <53 mmol/mol (<7%), whereas twice as many women had an HbA1c ≥53 mmol/mol (7%) with Standard Care (Standard Care; 20% vs. 10%) (Figure 2). At hospital discharge, costs for women with an HbA1c <53 mmol/mol (<7%) were about half those for women with an HbA1c ≥53 mmol/mol (≥7%).

Most of the savings (90%) were driven by the projected reduced use of intensive neonatal care (levels 1–3) among infants of mothers treated with CamAPS FX AID. Specifically, with CamAPS FX AID, length of stay in normal care increased by 5%, while length of stay in high‐dependency care (levels 1–2) and level 3 neonatal intensive care units decreased by 15.8% and 12.4%, respectively. This translated to cost reductions per 1000 women and singleton births of £490,887 for level 1–2 care and £463,485 for level 3 care. Additionally, a reduction of 12.8% in pre‐eclampsia cases was projected with CamAPS FX AID, resulting in less cost for the UK healthcare system by £92,725 per 1000 treated women. Respectively, reduced clinic visits resulted in a cost reduction of £83,808.

By third trimester maternal HbA1c level, the total costs per mother and infants, not including clinic visits, were estimated as follows: £12,133 for HbA1c <42 mmol/mol (<6%), £13,045 for HbA1c ≥42–53 mmol/mol (≥6%–<7%), £24,283 HbA1c ≥53 mmol/mol (≥7%).

### 3.2 Sensitivity analysis

The sensitivity analysis results suggest that pregnant women with T1D on CamAPS FX AID would be distributed in the HbA1c groups as follows: 62.8% in HbA1c <42 mmol/mol (<6%), 26.8% in HbA1c ≥42–53 mmol/mol (≥6%–<7%), 10.4% in HbA1c ≥53 mmol/mol (≥7%) with subsequent total costs estimated at £87,805,327. For the Standard Care, the respective costs were estimated at £94,491,666. Therefore, the potential cost savings to the UK healthcare system with CamAPS FX AID compared to Standard Care would be ‐£6,686,339. These results are in line with the base case analysis (Table 3).

**TABLE 3:** Modelled total NHS healthcare costs for pregnancies with type 1 diabetes in the UK (2021–2023), comparing CamAPS FX AID with Standard Care, stratified by third‐trimester HbA1c category.

Outcome (unit)	Standard care costs (£)	CamAPS FX costs (£)	Incremental costs (£)
Obstetric outcomes			
HbA1c <6%	£22,163,536	£26,523,575	£4,360,040
HbA1c ≥6%–<7%	£11,006,496	£10,662,543	−£343,953
HbA1c ≥7%	£8,764,864	£4,625,996	−£4,138,869
Neonatal outcomes			
HbA1c <6%	£19,883,915	£23,795,505	£3,911,590
HbA1c ≥6%–<7%	£12,707,770	£12,310,652	−£397,118
HbA1c ≥7%	£17,929,200	£8,393,168	−£9,536,032
Clinic visits	£2,071,105	£1,518,810	−£552,295
Total	£94,526,886	£87,830,250	−£6,696,636
Total PSA	£94,491,666	£87,805,327	−£6,686,339

*Note*: Incremental cost was calculated as CamAPS FX AID minus Standard Care; negative values indicate lower costs with CamAPS FX.

Abbreviation: PSA, Probabilistic sensitivity analysis.

Finally, across all the deterministic one‐way sensitivity analyses varying the cost inputs by ±20%, CamAPS FX resulted in incremental cost reduction ranging from −£6,049,647 to −£7,343,625 compared to Standard Care, indicating that the direction of cost reduction was not affected by any individual cost variation (Table S1). The two parameters with the greatest influence on the results were high dependency level care (level 1–2) and NICU (level 3), which produced impact ranges of £1,293,978 and £1,221,746, respectively, meaning that these two levels of care represent the largest absolute cost difference between the two treatment groups.

## 4 DISCUSSION

This analysis suggests that improved maternal glycaemia during type 1 diabetes pregnancy observed with CamAPS FX AID may translate into improved modelled obstetric and neonatal outcomes while reducing UK healthcare resource burden, with an estimated £6.7 M cost reduction between 2021 and 2023. To our knowledge, this is the first study to assess the economic impact of AID use during type 1 diabetes pregnancy in the United Kingdom. This builds on the prior work of Beato et al.^11^ by applying a similar modelling framework to a UK NHS setting. The additional contribution lies in the UK‐specific population estimates, NHS cost inputs and healthcare pathway assumptions.

In recent years, AID systems have demonstrated improved glycaemic outcomes across children, adolescents^21^ and adults with type 1 diabetes.^22^ In 2020^23^ NICE recommended the use of CGM during pregnancy and in 2023, based on the AiDAPT trial results, a NICE Technology Appraisal [TA 943] recommended offering AID systems to women with type 1 diabetes who are pregnant or planning to become pregnant, subject to cost‐effective procurement and implementation. Clear targets were set out by NHS England for the use of pregnancy‐specific AID systems ‘aiming for >60% of women in 2025/26 and >95% from 2026/27’. This is reinforced by the Saving Babies' Lives Care bundle, Version 3.2 (25/04/2025), which requires all maternity clinics to record and report their use of pregnancy‐specific hybrid closed‐loop systems. Pregnancy‐specific systems are defined as having a licence for use in pregnancy, a glucose target of ≤5.0 mmol/L and evidence of a clinically relevant improvement in maternal glucose outcomes (>5% increased time in the pregnancy glucose target range of 3.5–7.8 mmol/L compared to Standard Care). The CamAPS FX system is the only AID system currently fulfilling these criteria. Increased surveillance and additional healthcare support were advised for all pregnancies with third‐trimester HbA1c greater than 48 mmol/mol (6.5%).^24^

Many studies have examined the association between improved maternal HbA1c and reduced risk of maternal and neonatal complications.^15^, ^25^, ^26^ The AiDAPT trial was the first RCT to compare standard insulin delivery (pump or multiple daily injections) and CGM with a pregnancy‐specific AID system, demonstrating improved glycaemic outcomes with immediate benefits for mothers and babies.^18^ This contrasts with observational studies using other off‐label non‐pregnancy specific AID systems in pregnancy, which found no significant differences in clinical outcomes.^27^ The CamAPS FX algorithm incorporated pregnancy physiology and requirements as a key consideration from its early development stages^5^, ^6^ and notably its lower glucose targets and the adaptive ability of its algorithm may explain these differences.

Significant improvements in both the primary outcome (TIRp) and third‐trimester HbA1c observed with CamAPS FX AID provided a strong basis for incorporating AiDAPT trial data into the current economic model. With CamAPS FX AID, around 10% more women are projected to reach the recommended HbA1c target levels of <43 mmol/mol (6%). Better glycaemic control was associated with fewer maternal and neonatal complications within the modelled HbA1c framework used in this study, with most cost savings being driven by the reduced neonatal length of stay in intensive and high dependency care units. Avoiding high‐level neonatal admissions may not only reduce projected direct costs but could also free critical care capacity, optimising both clinical and resource outcomes.

Although the studies differ in regions, population characteristics and clinical results, our findings are in line with those of Azahaf et al., highlighting potential cost‐savings with off‐label use of the MiniMed 780G AID system.^12^ They employed a different modelling approach and due to the lack of significant findings for the primary outcome (TIRp), analyses were limited to estimating direct healthcare costs.

A key strength of our study is the use of high‐quality data from the AiDAPT trial with standardised systematic data collection, minimising bias and allowing for detailed between‐group comparisons. The data reflect UK‐healthcare settings, aligning with the most recent NHS cost references, making our findings highly relevant for informing decision‐making in a resource‐constrained healthcare system. Another strength is that we show healthcare expenditure at hospital discharge for each third trimester HbA1c category, meaning that our model estimates the potential impact on the NHS healthcare system, according to maternal glycaemia. Additionally, we presented costs and savings both per 1000 pregnancies and at the population level, offering insights into the economic implications of adopting CamAPS FX AID nationwide.

This study also has limitations. Most importantly, the AiDAPT trial was not powered to detect differences in obstetric and neonatal outcomes. To address this, we collectively assessed the glycaemic control association with the independent outcomes rather than direct causal effects. Additionally, delivery costs among women with pre‐eclampsia were not modelled by delivery mode, but subsequent delivery was assumed to occur and was costed according to Fox et al.^19^ Although this approach did not allow for delivery pathway differentiation in women with pre‐eclampsia, it reflects the data limitations and avoids the over‐parameterisation of the model. Pre‐eclampsia costs were derived from an Irish publication and considered reasonably applicable to the UK setting, given the similarities between the Irish and UK healthcare systems in terms of clinical pathways and publicly funded care structures. Also, data availability restricted the inclusion of additional cost factors: birth‐related injuries, maternal postpartum care, neonatal readmissions, long‐term outcomes or any costs associated with clinical negligence claims or compensation related to adverse events. All of which are a substantial financial burden within the NHS,^28^ meaning that the broader economic impact of improved glycaemic management is likely underestimated. Hence, the length of stay costs may not reflect the full cost of neonatal complications or any lifelong impacts. These omissions likely underestimate the modelled cost‐impact associated with HbA1c improvement observed with CamAPS FX AID use, since HbA1c was used as a modelling variable, potentially not fully capturing the glycaemic benefits with CamAPS FX AID, including TIRp, hyperglycaemia exposure and glycaemic variability.

A cost‐effectiveness analysis was not feasible since quality‐of‐life data were only collected for mothers and not for neonates. Since neonatal outcomes contributed substantially to both health outcomes and associated costs, this was inapplicable. Furthermore, as device acquisition and training costs were not included, our analysis represents a partial cost‐consequence assessment focusing on the neonatal and obstetric outcomes. Given that device and training costs vary across AID systems and healthcare settings, the generalisability of the findings to other AID systems depends on the ability to achieve comparable glycaemic control improvements. Therefore, the current model provides a comprehensive albeit conservative cost assessment based on the available immediate obstetric and neonatal complications related to improved glycaemic control during type 1 diabetes pregnancy.

Future research should explore additional outcomes, including long‐term maternal and child health, pre‐pregnancy and postpartum periods, as well as neonate's health‐related quality of life estimates. For example, pre‐eclampsia is not only associated with fetal growth restriction,^29^ higher risk of hypoxic ischemic encephalopathy, sepsis and perinatal death,^30^ but also with increased risk for maternal cardiovascular disease in later life.^31^, ^32^

Achieving optimal glycaemic control before and during pregnancy is critical, as women entering pregnancy with HbA1c levels above target have a significantly higher risk of complications.^2^ CamAPS FX AID may serve as a valuable tool not only during pregnancy^18^ and post‐partum sustaining glycaemic outcomes,^33^ but also when planning pregnancy, where optimising early glycaemic control reduces major congenital anomalies.^2^ Thus, future research should explore the economic impact of initiating CamAPS FX AID preconception, which could yield additional clinical and economic benefits.

## 5 CONCLUSION

This study provides the first model‐based health economic evaluation of a pregnancy‐specific AID system for pregnant women with type 1 diabetes in the United Kingdom. Using the AiDAPT trial data, the model projected that HbA1c improvement associated with the use of CamAPS FX AID during pregnancy would shift more women into lower HbA1c categories, with associated reduced neonatal care needs. These modelled clinical benefits translated into substantial projected cost savings for the UK healthcare system—estimated at £1000 per T1D pregnancy, primarily driven by the lower severity level of neonatal care needed.

Grounded in the AiDAPT trial data, our findings fully support the NHS England and Saving Babies Lives recommendations and provide model‐based evidence to highlight the value of expanding access to pregnancy‐specific AID systems during pregnancy. Further research incorporating long‐term maternal and neonatal outcomes, medico‐legal litigation costs and quality of life data could better estimate the true cost of suboptimal glycaemic levels during type 1 diabetes pregnancy.

## AUTHOR CONTRIBUTIONS

Conceptualisation: MES, AKM, TL, ES, HM, Study design and methodology: MES, AKM, Data Collection and resources: MES, TL, HM, Formal analysis: MES, TL, Results interpretation: AKM, MES, TL, ES, FC, HM, Original draft writing: MES, Critical input and revision of the manuscript: MES, AKM, TL, ES, FC, HM, Project supervision: MES, AKM, HM.

## FUNDING INFORMATION

The AiDAPT trial was funded by the Efficacy and Mechanism Evaluation Programme (NIHR EME reference 16/35/01) an MRC and NIHR partnership. The views expressed in this publication are those of the author(s) and not necessarily those of the MRC, NIHR or the Department of Health and Social Care. Two co‐authors (MES, AKM) are employees of Mylife Diabetes Care AG, who provided additional health economic expertise for the analyses.

## CONFLICT OF INTEREST STATEMENT

AKM, MES are employees of mylife Diabetes Care AG. EMS has received research funding from NIHR, Medical Research Council and Abbott Diabetes Care; EMS has received speaker fees and expenses from Abbott Diabetes Care, Eli Lilly Diabetes Care and Ypsomed Diabetes Care. HRM serves on the Medtronic European and the Ypsomed Scientific Advisory Boards, reports research devices from Abbott Diabetes Care (no cost), Dexcom (reduced cost) and Medtronic (no cost), speaker honoraria from Abbott Diabetes Care, Dexcom, Eli Lilly, Medtronic, Novo Nordisk, Sanofi and Ypsomed. She chairs the National Pregnancy in Diabetes (NPID) audit and is a member of the editorial boards for Diabetes Care and Diabetologia journals.

## ETHICS STATEMENT

Ethical approval for the health economic evaluation was obtained from the East of England Research Ethics Committee (Cambridge Central Research Ethics Committee reference 18/EE/0084) and approved by the Health Regulatory Agency (HRA) for participating NHS organisations in England and by NHS organisations in Scotland and Northern Ireland. Each participant signed a consent form prior to participating in the study.

## Supporting information


**Table S1.** Deterministic one‐way sensitivity analysis results, modelling the total NHS healthcare costs for pregnant women with type 1 diabetes in the United Kingdom based on UK national audit data (2021–2023). Standard Care is compared with CamAPS FX AID. All costs are in 2024 £.

**Data S1:** CHEERS 2022 Checklist.

## Data Availability

Requests for access to trial data will be considered and approved in writing where appropriate, after formal application to the TSC. Inquiries for data should be addressed to the principal investigator Prof Helen R. Murphy.

## References

[1] Holmes VA , Young IS , Patterson CC , et al. Optimal glycemic control, pre‐eclampsia, and gestational hypertension in women with type 1 diabetes in the diabetes and pre‐eclampsia intervention trial. Diabetes Care. 2011;34(8):1683‐1688. doi:10.2337/dc11-0244 21636798 PMC3142058

[2] Murphy HR , Howgate C , O'Keefe J , et al. Characteristics and outcomes of pregnant women with type 1 or type 2 diabetes: a 5‐year national population‐based cohort study. Lancet Diabetes Endocrinol. 2021;9(3):153‐164. doi:10.1016/s2213-8587(20)30406-x 33516295

[3] Tundidor D , Meek CL , Yamamoto J , et al. Continuous glucose monitoring time‐in‐range and HbA1c targets in pregnant women with type 1 diabetes. Diabetes Technol Ther. 2021;23(10):710‐714. doi:10.1089/dia.2021.0073 33945304 PMC8573793

[4] Benhalima K , Jendle J , Beunen K , Ringholm L . Automated insulin delivery for pregnant women with type 1 diabetes: where do we stand? J Diabetes Sci Technol. 2024;18(6):1334‐1345. doi:10.1177/19322968231223934 38197363 PMC11535386

[5] Stewart ZA , Wilinska ME , Hartnell S , et al. Closed‐loop insulin delivery during pregnancy in women with type 1 diabetes. N Engl J Med Overseas Ed. 2016;375(7):644‐654. doi:10.1056/NEJMoa1602494 27532830

[6] Stewart ZA , Wilinska ME , Hartnell S , et al. Day‐and‐night closed‐loop insulin delivery in a broad population of pregnant women with type 1 diabetes: a randomized controlled crossover trial. Diabetes Care. 2018;41(7):1391‐1399. doi:10.2337/dc17-2534 29535135

[7] Quirós C , Herrera MT , Amigó J , et al. Real‐world evidence of off‐label use of commercially automated insulin delivery systems compared to multiple daily insulin injections in pregnancies complicated by type 1 diabetes. Diabetes Technol Ther. 2024;26(8):596‐606. doi:10.1089/dia.2023.0594 38417014

[8] Lee TTM , Collett C , Bergford S , et al. Automated insulin delivery in women with pregnancy complicated by type 1 diabetes. N Engl J Med Overseas Ed. 2023;389(17):1566‐1578. doi:10.1056/NEJMoa2303911 37796241

[9] Benhalima K , Beunen K , Wilder NV , et al. Comparing advanced hybrid closed loop therapy and standard insulin therapy in pregnant women with type 1 diabetes (CRISTAL): a parallel‐group, open‐label, randomised controlled trial. Lancet Diabetes Endocrinol. 2024;12(6):390‐403. doi:10.1016/s2213-8587(24)00089-5 38697182

[10] Perea V , Quirós C , Herrera‐Arranz MT , et al. Pregnancy outcomes with the pregestational use of Minimed 780G compared to Minimed 640G: findings from a multicenter cohort study. Acta Diabetol. 2024;62:1117. doi:10.1007/s00592-024-02430-x 39630235

[11] Beato‐Víbora PI , Syleouni M‐E , Lee TTM , et al. Optimizing diabetes management during type 1 diabetes pregnancy with automated insulin delivery therapy: clinical impact and economic consequences in Spain. Diabetes Technol Ther. 2026;15209156261423217. doi:10.1177/15209156261423217 41703432

[12] Azahaf S , Beunen K , Wilder NV , et al. Cost‐effectiveness of advanced hybrid closed loop therapy compared to standard insulin therapy for type 1 diabetes in pregnancy: an economic evaluation of the CRISTAL trial. EClinicalMedicine. 2025;81:103106. doi:10.1016/j.eclinm.2025.103106 40034575 PMC11874532

[13] Gribble KD , Bewley S , Bartick MC , et al. Effective communication about pregnancy, birth, lactation, breastfeeding and newborn care: the importance of sexed language. Front Glob Womens Health. 2022;3:818856. doi:10.3389/fgwh.2022.818856 PMC8864964 35224545

[14] NHS . “National pregnancy in diabetes audit 2021–2023 dashboard 2023.” Accessed July 17, 2025. https://digital.nhs.uk/data‐and‐information/publications/statistical/national‐pregnancy‐in‐diabetes‐audit/2023

[15] Lemaitre M , Ternynck C , Bourry J , Baudoux F , Subtil D , Vambergue A . Association between HbA1c levels on adverse pregnancy outcomes during pregnancy in patients with type 1 diabetes. J Clin Endocrinol Metab. 2022;107(3):e1117‐e1125. doi:10.1210/clinem/dgab769 34694409 PMC8852207

[16] NICE . “Diabetes in pregnancy: Management from preconception to the postnatal period. NICE guideline.” www.nice.org.uk/guidance/ng3 32212588

[17] England N . National pregnancy in diabetes audit report. NHS England; 2020. https://digital.nhs.uk/data‐and‐information/publications/statistical/national‐pregnancy‐in‐diabetes‐audit/2019‐and‐2020

[18] Lee TT , Collett C , Bergford S , et al. Automated closed‐loop insulin delivery for the management of type 1 diabetes during pregnancy: the AiDAPT RCT. Efficacy and Mechanism Evaluation, No. 11.07. 2024:1‐80. doi:10.3310/WCHZ4201 38718153

[19] Fox A , McHugh S , Browne J , et al. Estimating the cost of preeclampsia in the healthcare system. Hypertension. 2017;70(6):1243‐1249. doi:10.1161/HYPERTENSIONAHA.117.09499 29084880

[20] England N . “National Cost Collection: National schedule of NHS costs ‐ Year 2022/23 ‐ NHS trusts and NHS foundation trusts.” 2024 https://www.england.nhs.uk/publication/2022‐23‐national‐cost‐collection‐data‐publication/

[21] Ng SM , Wright NP , Yardley D , et al. Long‐term assessment of the NHS hybrid closed‐loop real‐world study on glycaemic outcomes, time‐in‐range, and quality of life in children and young people with type 1 diabetes. BMC Med. 2024;22(1):175. doi:10.1186/s12916-024-03396-x 38659016 PMC11044460

[22] Stahl‐Pehe A , Shokri‐Mashhadi N , Wirth M , et al. Efficacy of automated insulin delivery systems in people with type 1 diabetes: a systematic review and network meta‐analysis of outpatient randomised controlled trials. EClinicalMedicine. 2025;82:103190. doi:10.1016/j.eclinm.2025.103190 40270713 PMC12017971

[23] Murphy HR . NICE guideline update: good news for pregnant women with type 1 diabetes and past or current gestational diabetes. Diabet Med. 2020;38(6):e14576. doi:10.1111/dme.14576 33793978

[24] England N . “Saving babies' lives: version 3.” 2025. https://www.england.nhs.uk/long‐read/saving‐babies‐lives‐version‐3‐2/

[25] Feig DS , Donovan LE , Corcoy R , Murphy KE , Amiel SA . Continuous glucose monitoring in pregnant women with type 1 diabetes (CONCEPTT): a multicentre international randomized controlled trial. Obstet Anesth Dig. 2018;38(3):139. doi:10.1097/01.aoa.0000542359.79329.36 PMC5713979 28923465

[26] M EI . Risk of complications of pregnancy in women with type 1 diabetes: nationwide prospective study in The Netherlands. BMJ. 2004;328(7445):915. doi:10.1136/bmj.38043.583160.EE 15066886 PMC390158

[27] Sobhani NC . Impact of AID on glycemic profile and maternal/neonatal outcomes in pregnancy: a review of the evidence from observational studies. J Diabetes Sci Technol. 2025;20(1):7‐14. doi:10.1177/19322968251327603 40119663 PMC11948270

[28] Limb M . NHS medical negligence costs reach £60bn as maternity payouts soar. BMJ. 2025;391:r2211. doi:10.1136/bmj.r2211 41115705

[29] Odegård RA , Vatten LJ , Nilsen ST , Salvesen KA , Austgulen R . Preeclampsia and fetal growth. Obstet Gynecol. 2000;96(6):950‐955. 11084184

[30] Ulfsdottir H , Grandahl M , Björk J , Karlemark S , Ekéus C . The association between pre‐eclampsia and neonatal complications in relation to gestational age. Acta Paediatr Suppl. 2024;113(3):426‐433. doi:10.1111/apa.17080 38140818

[31] Pittara T , Vyrides A , Lamnisos D , Giannakou K . Pre‐eclampsia and long‐term health outcomes for mother and infant: an umbrella review. BJOG. 2021;128(9):1421‐1430. doi:10.1111/1471-0528.16683 33638891

[32] Berks D , Hoedjes M , Raat H , Duvekot J , Steegers E , Habbema J . Risk of cardiovascular disease after pre‐eclampsia and the effect of lifestyle interventions: a literature‐based study. BJOG. 2013;120(8):924‐931. doi:10.1111/1471-0528.12191 23530583

[33] Lee TTM , Collett C , Bergford S , et al. Automated insulin delivery during the first 6 months postpartum (AiDAPT): a prespecified extension study. Lancet Diabetes Endocrinol. 2025;13(3):210‐220. doi:10.1016/S2213-8587(24)00340-1 39884300 PMC7617783

