# Supplementary material for: HbA1c improvement in pregnant women with type 1 diabetes using the CamAPS FX automated insulin delivery (AID) system: Clinical and economic outcomes
Source: Diabet Med. 2026 Jun 21;43(8):e70394. doi: 10.1111/dme.70394 (PMC13380392; doi:10.1111/dme.70394)
Supplement: Supplementary file 1 — Table S1. Deterministic one‐way sensitivity analysis results, modelling the total NHS healthcare costs for pregnant women with type 1 diabetes in the United Kingdom based on UK national audit data (2021–2023). Standard Care is compared with CamAPS FX AID. All costs are in 2024 £. [file DME-43-e70394-s001.docx]

## Supplementary Table 1. Deterministic one-way sensitivity analysis results, modelling the total NHS healthcare costs for pregnant women with type 1 diabetes in the UK based on UK national audit data (2021-2023). Standard Care is compared with CamAPS FX AID. All costs are in 2024 £.

| **Varying cost parameter** | **Incremental savings** | **Base-case incremental savings** | **Impact Range** |
| --- | --- | --- | --- |
| **Obstetric outcomes** |  |  |  |
| **Preeclampsia including subsequent delivery** |  |  | £244,422 |
| Scenario +20% | **£6,818,847** | £6,696,636 |  |
| Scenario -20% | £6,574,425 | £6,696,636 |  |
| **Vaginal delivery** |  |  | £80,629 |
| Scenario +20% | £6,656,322 | £6,696,636 |  |
| Scenario -20% | **£6,736,950** | £6,696,636 |  |
| **C-section** |  |  | £114,681 |
| Scenario +20% | £6,639,296 | £6,696,636 |  |
| Scenario -20% | **£6,753,977** | £6,696,636 |  |
| **Neonatal outcomes** |  |  |  |
| **Normal care** |  |  | £107,100 |
| Scenario +20% | £6,643,086 | £6,696,636 |  |
| Scenario -20% | **£6,750,186** | £6,696,636 |  |
| **High dependency level 1-2** |  |  | £1,293,978 |
| Scenario +20% | **£7,343,625** | £6,696,636 |  |
| Scenario -20% | £6,049,647 | £6,696,636 |  |
| **Neonatal intensive care unit level 3** |  |  | £1,221,746 |
| Scenario +20% | **£7,307,509** | £6,696,636 |  |
| Scenario -20% | £6,085,763 | £6,696,636 |  |
| **Clinic visits** |  |  | £220,918 |
| Scenario +20% | **£6,807,095** | £6,696,636 |  |
| Scenario -20% | £6,586,177 | £6,696,636 |  |

*Incremental cost was calculated as Standard Care minus CamAPS FX AID. Bold values indicate that incremental costs savings exceed base case. Impact range is estimated as saving at +20% minus saving at -20%, reflecting the influence of the cost parameter on the overall result.*
